# Extracorporeal Life Support Increases Survival After Prolonged Ventricular Fibrillation Cardiac Arrest in the Rat

**DOI:** 10.1097/SHK.0000000000000909

**Published:** 2017-11-13

**Authors:** Ingrid Anna Maria Magnet, Florian Ettl, Andreas Schober, Alexandra-Maria Warenits, Daniel Grassmann, Michael Wagner, Christoph Schriefl, Christian Clodi, Ursula Teubenbacher, Sandra Högler, Wolfgang Weihs, Fritz Sterz, Andreas Janata

**Affiliations:** ∗ Department of Emergency Medicine, Medical University of Vienna, Vienna, Austria; † University of Veterinary Medicine Vienna, Vienna, Austria; ‡ Division of Biomedical Research, Medical University of Vienna, Vienna, Austria

**Keywords:** Animal models, cardiopulmonary bypass, cardiopulmonary resuscitation, ischemia, reperfusion

## Abstract

**Background:**

Extracorporeal life support (ECLS) for cardiopulmonary resuscitation (CPR) may increase end organ perfusion and thus survival when conventional CPR fails. The aim was to investigate, if after ventricular fibrillation cardiac arrest in rodents ECLS improves outcome compared with conventional CPR.

**Methods:**

In 24 adult male Sprague–Dawley rats (460–510 g) resuscitation was started after 10 min of no-flow with ECLS (consisting of an open reservoir, roller pump, and membrane oxygenator, connected to cannulas in the jugular vein and femoral artery, n = 8) or CPR (mechanical chest compressions plus ventilations, n = 8) and compared with a sham group (n = 8). After return of spontaneous circulation (ROSC), all rats were maintained at 33°C for 12 h. Survival to 14 days, neurologic deficit scores and overall performance categories were assessed.

**Results:**

ECLS leads to sustained ROSC in 8 of 8 (100%) and neurological intact survival to 14 days in 7 of 8 rats (88%), compared with 5 of 8 (63%) and 1 of 8 CPR rats. The median survival time was 14 days (IQR: 14–14) in the ECLS and 1 day (IQR: 0 to 5) for the CPR group (*P* = 0.004).

**Conclusion:**

In a rat model of prolonged ventricular fibrillation cardiac arrest, ECLS with mild hypothermia produces 100% resuscitability and 88% long-term survival, significantly better than conventional CPR.

## INTRODUCTION

### Background

Extracorporeal life support (ECLS) for cardiopulmonary resuscitation (CPR) has been increasingly used as a rescue therapy for patients in cardiac arrest (CA) refractory to conventional measures of CPR (1). Experimental research showed that cerebral perfusion by conventional CPR is poor after increasing no- and low-flow times (2, 3). ECLS provides vital organ perfusion over longer periods, improving resuscitability and neurologically intact survival compared with prolonged standard CPR (4). ECLS thus facilitates treatment of reversible causes of CA, like coronary intervention in acute myocardial infarction (5, 6). ECLS also allows control of reperfusion conditions to counteract reperfusion injury, such as reperfusate temperature for intra-arrest cooling (7, 8).

Animal studies are required to devise this therapeutic concept to its fullest potential for saving human lives in a bench to bedside approach. Large animal models of ECLS (e.g., dogs or swine) mimic the clinical setting best, but are cost- and labor-intensive (9, 10). A small rodent model of ventricular fibrillation (VF) CA with ECLS, replacing and reducing the use of large experimental animals, and refining outcome measures by introducing molecular and immunohistochemical tools to investigate mechanisms of ischemic and reperfusion damage, was only recently developed (11). It was shown that ECLS after a 6-min VFCA in a rat was feasible, but did not improve survival, neurologic or histologic outcome versus conventional CPR. The authors concluded this was due to the short duration of the ischemic insult, and that further studies of ECLS in rats with longer VFCA times were needed.

### Objectives

It was the aim of this study to assess the feasibility of ECLS following a prolonged global ischemic insult of 10 min VFCA in the rat. We hypothesized that with prolonged insult times, ECLS compared with conventional CPR would improve long-term survival, neurologic and histologic outcome.

## METHODS

### Study design and ethical statement

This was a non-randomized controlled laboratory study of ECLS versus CPR in a rodent VFCA model. The experimental protocol was approved by the Institutional Animal Care and Use Committee of the Medical University of Vienna and the Austrian Federal Ministry of Science, Research and Economy (GZ.: 66.009/0064-II/3b/2011) following the ARRIVE (12) and Directive 2010/63/EU guidelines. Twenty-four adult male Sprague–Dawley rats (460– 510 g) were included in this study, 16 of which underwent VFCA; the first eight were resuscitated by ECLS, followed by eight rats with conventional CPR. Eight rats served as naive histologic controls. Lack of randomization to the treatment arm was for logistic reasons, since setup of the ECLS required considerable preparatory work, in order to perform all ECLS experiments within 2 weeks, followed by 2 weeks of CPR experiments. Animal care and handling and all experiments were performed at the Division of Biomedical Research, where rats are held in small groups in standard rodent housing with soft wood bedding, at room temperature (22 ± 2°C, humidity 55 ± 10%), on a 12 h light/dark cycle, with unrestricted access to food and water.

### Animal preparation

**Fig. 1:**
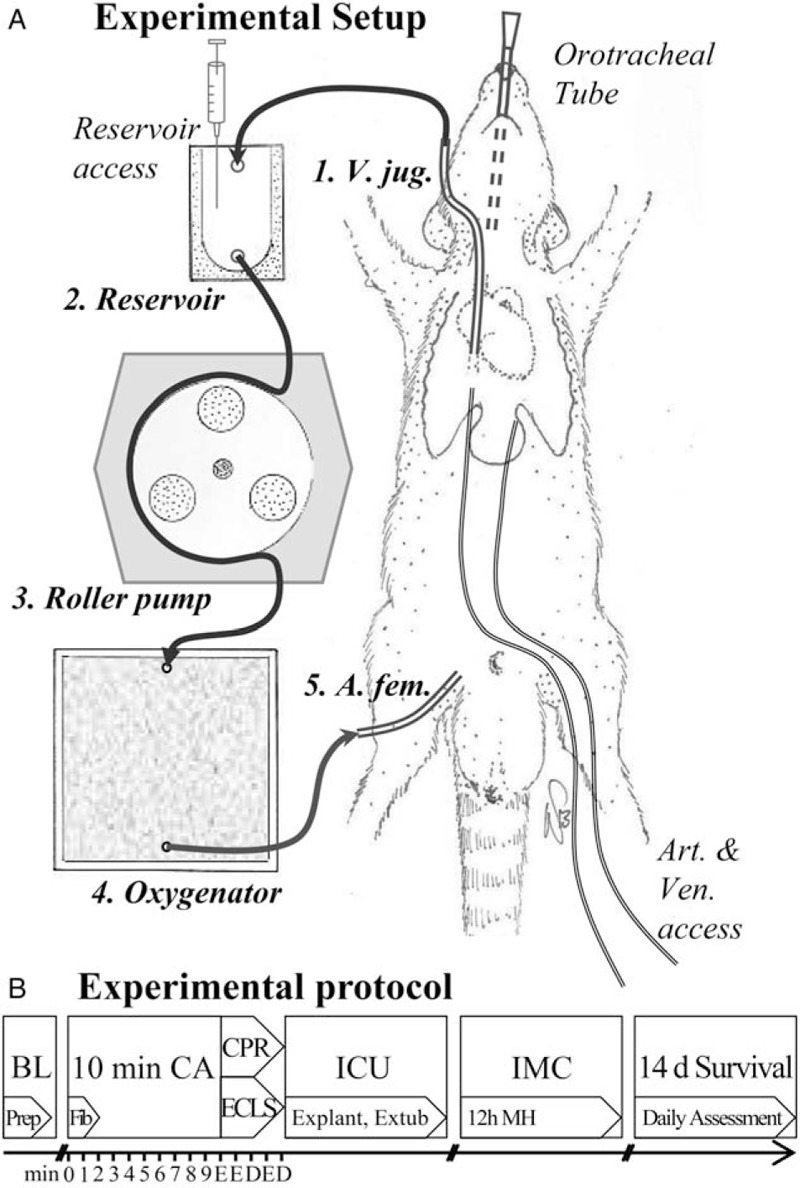
The ECLS setup (A) consisted of an open reservoir (2), a roller pump (3), a membrane oxygenator (4), connected by silicone tubing to cannulas in the right jugular vein (1) and right femoral artery (5), primed with 15 mL of crystalloid solution and temperature controlled at 33°C by a circulating water bath (not depicted).

Following insufflation of 6% sevoflurane in oxygen and subcutaneous injection of buprenorphine (50 μg/kg body weight, BW), rats were orotracheally intubated with a 14-gauge intravenous cannula (Venflon BD Luer-Lok, Helsingborg, Sweden) and volume-controlled ventilated (tidal volume 7 cc/kg BW, inspiration to expiration ratio 1:2, ventilation rate 65/min; Inspira advanced safety ventilator, Harvard Apparatus, Holliston, Mass). During preparation, sevoflurane 3.5% and FiO_2_ 0.5 were maintained, then reduced to sevoflurane 2.5% and FiO2 0.3. Three-lead electrocardiogram (ECG), end-tidal CO_2_ (etCO_2_; FilterLine Set Microstream, Oridion Capnography, Needham, Mass) and rectal temperature probes (T_rec_; Mon-a-therm 9-french thermistor probe, Mallinckrodt Medical, St. Louis, Mo) were placed. Baseline temperature was held at 37 ± 0.5°C with a heated small animal operating table (Medax, Neumünster, Germany). Using aseptic techniques, catheters (Argyle 2.5-french umbilical vessel catheter, Covidien, Dublin, Ireland) were inserted 9 and 11 cm into the left femoral vein and artery for hemodynamic monitoring, drug administration, and arterial blood sampling. In the ECLS group, custom cannulas were inserted into the right femoral artery (approximately 22-gauge, 2-cm-long) and right jugular vein (approximately 14-gauge, 5-cm-long with multiple openings). For induction of VFCA, a pacing catheter (3-french Bi-Pacing-Ball, Vygon, Ecouen, France) was inserted 5 cm into the venous ECLS cannula or the right jugular vein. Adhesive defibrillation paddles were fixated on the lateral thorax and connected to a biphasic manual defibrillator (HeartStart MRx ALS Monitor/Defibrillator, Philips). After preparation, heparin (500 IU/kg BW) was administered in both groups to avoid clotting of the cannulas. The custom-made ECLS setup (Dipl.Ing. Martin Humbs, Valley, Germany) as previously described (11) and depicted in Figure 1 consisted of an open reservoir, a capillary membrane oxygenator from three-layered micro-porous polypropylene, and a roller pump (Masterflex L/S PTFE Tubing Pump, Cole-Parmer, Vernon Hills, Ill), connected by silicone tubing to the ECLS cannulas. The circuit was primed with 15 mL balanced crystalloid solution (Elo-Mel isoton Infusionlösung—Na 140, K 5.0, Ca 2.5, magnesium 1.5, chloride 108, acetate 45 mmol/L, Fresenius Kabi, Graz-Puntigam, Austria) and temperature controlled at 33°C (Refrigerated/Heated Circulator Water Bath 1166D, VWR Polyscience, Niles, Ill). Sweep gas flow to the oxygenator was 400 mL/min with 95% O_2_ and 5% CO_2_, producing normocapnia and a pO_2_ greater 400 mm Hg postoxygenator.

Experimental protocol (B): BL indicates baseline, preparation (Prep) with intubation and insertion of all catheters; CA, cardiac arrest, alternating current fibrillation (Fib); CPR, cardiopulmonary resuscitation; D, defibrillation; ECLS, extracorporeal life support; E, epinephrine dose; ICU, intensive care, explantation of all catheters, weaning from ventilator and extubation, implantation of temperature probe; IMC, intermediate care with supplemental oxygen and 12 h of mild hypothermia (MH) before slow rewarming.

### Study protocol

Ninety seconds before VFCA, sevoflurane was turned off (Fig. 1). A 2-min 50 Hz/5 mA alternating electrical current induced VFCA, repeated for 30 s if spontaneous defibrillation occurred. ECG and arterial blood pressure reading confirmed VFCA, the pacing catheter was removed and the orotracheal tube disconnected. After 10 min of no-flow, resuscitation started with mechanical ventilation (20/min, FiO_2_ 1.0) and either ECLS at a flow rate of 100 mL/kg BW as feasible by passive venous return, or chest compressions of 200/min delivered with a pneumatic small animal chest compression device (Streubel Automation, Grampersdorf, Germany). Epinephrine (20 μg/kg BW), sodium bicarbonate (1 mmol/kg BW), and heparin (200 IU/kg BW) were given 15 s before resuscitation into the reservoir or intravenously; epinephrine 10 μg/kg BW administration was repeated 60 s after the start of resuscitation and every 2 min thereafter. Defibrillation (2 shocks/5 Joule) was attempted at 2 min of resuscitation, and every 2 min thereafter. After ROSC—an organized cardiac rhythm with pulsatile arterial pressure readings—rats were immediately weaned from ECLS or chest compressions stopped. Resuscitation was attempted 10 min maximum and not reinitiated if rearrest occurred. Sustained ROSC was defined as ≥20 min of spontaneous circulation with MAP ≥ 50 mm Hg.

After ROSC, ventilation rate was returned to 65/min, FiO_2_ reduced to 0.5, and buprenorphine injected subcutaneously. If MAP dropped below 60 mm Hg, crystalloid fluid boluses were given intravenously up to a maximum positive fluid balance of 10 mL. By 30 min, all cannulas were removed, the respective vessels ligated and skin incisions closed. A temperature monitoring telemetric probe was implanted into the peritoneal cavity (Mini-Mitter Co, Sunriver, Ore). Animals were weaned from ventilator, extubated, and placed in cages with supplemental oxygen. Temperature was controlled at 33°C for 12 h with a custom heating/fan system regulated by the implanted telemetry probe, then slowly rewarmed to 37°C. After 14 days or if humane endpoint was met according to laboratory animal ethical requirements (>20% loss of pre-experiment body weight, and/or severe distress or pain not relievable by supportive therapy) (13, 14), rats were euthanized with a sevoflurane and potassium overdose for histologic evaluation.

### Experimental outcomes

Primary outcome was survival to 14 days. Secondary outcome included neurologic and histopathologic evaluation. During the experiment, mean arterial pressure (MAP), etCO_2_ and temperature were continuously monitored and recorded (IntelliVue MP70 patient monitor, Philips, DA Best, The Netherlands). Arterial blood sampling was performed 5 min before VFCA, 5 and 15 min after ROSC. Measurements included partial oxygen (pO_2_) and carbon dioxide (pCO_2_) tension, pH, base excess (BE), hydrogen bicarbonate (HCO_3_), lactate (Lac), sodium (Na), potassium (K), calcium (Ca), hematocrit (Hct), and glucose (Glu).

Neurologic function was assessed daily with a 5-point overall performance category score (OPC; 1 = normal, 2 = slight disability, 3 = severe disability, 4 = comatose, 5 = dead) and a neurologic deficit score (NDS; 0 = normal, 100 = dead) (15). If animals did not drink and lost weight, 10 mL crystalloid fluid were injected subcutaneously. Buprenorphine was administered subcutaneously up to twice daily in signs of distress (16, 17). All neurologic evaluation was performed according to protocol by veterinarian Wolfgang Weihs.

For histologic evaluation, fluid-perfused, formalin-fixed brains were cut into 3-μm coronary sections between bregma 3.5 and 4.0 to depict the hippocampal CA1 region. Serial sections were stained with hematoxylin and eosin (HE), cresyl violet (Nissl), Fluoro-Jade-B (FJB; Merck Millipore, Darmstadt, Germany), and immunohistochemically with an antibody to ionized calcium-binding adaptor molecule 1 (Iba1; Wako Chemicals GmbH, Neuss, Germany). In HE- and Nissl-staining, viable neurons with a visible nucleolus were counted in two 250-μm sectors of the medial and lateral CA1 region at 200-fold magnification. In FJB staining, fluorescent dying neurons were rated semiquantitatively on a 5-point scale (0 = no fluorescence, 4 = intense fluorescence in the entire CA1 region). In Iba1-stained sections, microglial cells were identified and assessed semiquantitatively on a 5-point scale (0 = normal mild staining, 4 = intense staining in the entire CA1 region). All the histologic evaluations were performed blinded by veterinarian histopathologist Sandra Högler.

### Statistical methods

Continuous data distributed normally are reported as mean and standard deviation (SD). Semiquantitative evaluation of histologic FJB and Iba1 staining is reported as median and interquartile range (IQR). Group comparisons between ECLS and CPR or ECLS and naive were made with independent sample *t* test or Mann–Whitney *U* test (exact significance). Weight comparison between ECLS, CPR, and naive was performed with one-way analysis of variance. Discrete data are reported as counts and percentages and group comparisons were made with Fisher exact test. Survival time over 14 days is reported as median and IQR and plotted as Kaplan–Meier curve; group comparison was made with log-rank test. All statistical tests were two-sided and a *P* < 0.05 was considered significant. All calculations were performed with PASW Statistics for Windows 22.0 (SPSS, IBM Corporation, Armonk, NY).

## RESULTS

### Baseline data and adverse events

Rats weighed 471 ± 19 g in the ECLS, 499 ± 33 g in the CPR, and 481 ± 55 g in the naive group (*P* = non-significant, ns). Sevoflurane exposure before VFCA was equal in both groups (ECLS: 122 ± 26 min, CPR: 113 ± 18 min, *P* = ns), though insertion of cannulas took longer in the ECLS group (ECLS: 62 ± 17 min, CPR: 45 ± 10 min, *P* = 0.033), as did subsequent decannulation and extubation (ECLS: 55 ± 9 min, CPR: 35 ± 9 min, *P* = 0.004).

Four experiments failed because of technical difficulties and were redone: three rats suffered uncontrollable bleeding during preparation (two CPR, one ECLS), one died from ventilator failure after successful resuscitation (ECLS).

### Resuscitation

ROSC was achieved within 3 min in both groups (ECLS: 2.9 ± 1.0 min, CPR: 2.9 ± 0.8 min), requiring equal amounts of epinephrine (ECLS: 34 ± 5 μg/kg BW, CPR: 33 ± 5 μg/kg BW) and defibrillations (ECLS: 2.4 ± 1.1, CPR: 1.6 ± 0.7; all *P* = ns).

**Fig. 2:**
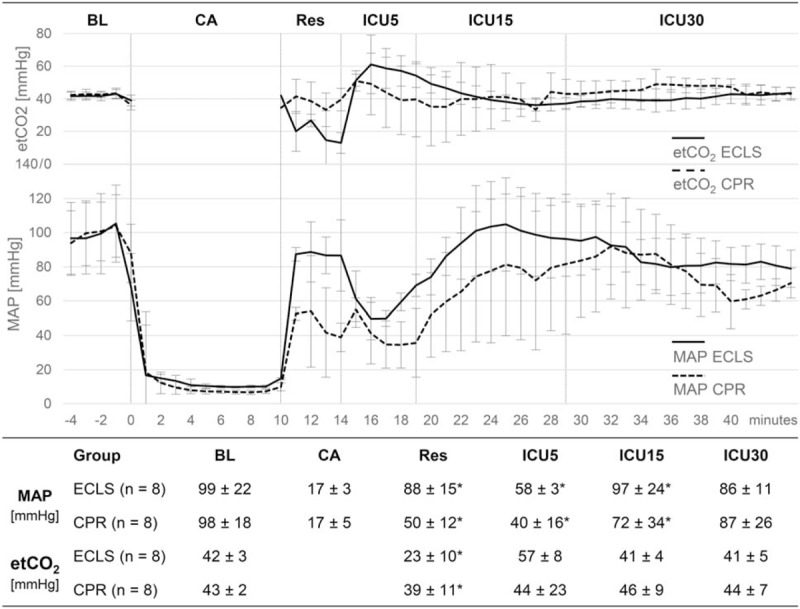
Hemodynamic data, including mean arterial pressure (MAP) and end-tidal CO_2_ (etCO_2_), are depicted over time (minutes) before baseline (BL) and after cardiac arrest (CA; start cardiac arrest = minute 0) and presented as mean and standard deviation.

ICU5, first 5 min after return of spontaneous circulation (ROSC); ICU15, minutes 6 to 15 after ROSC (n = 5 in the CPR group); ICU30, minutes 16 to 30 after ROSC (n = 5 in the CPR group); Res, resuscitation phase. ^∗^*P* < 0.05 comparing ECLS versus CPR.

**Fig. 3:**
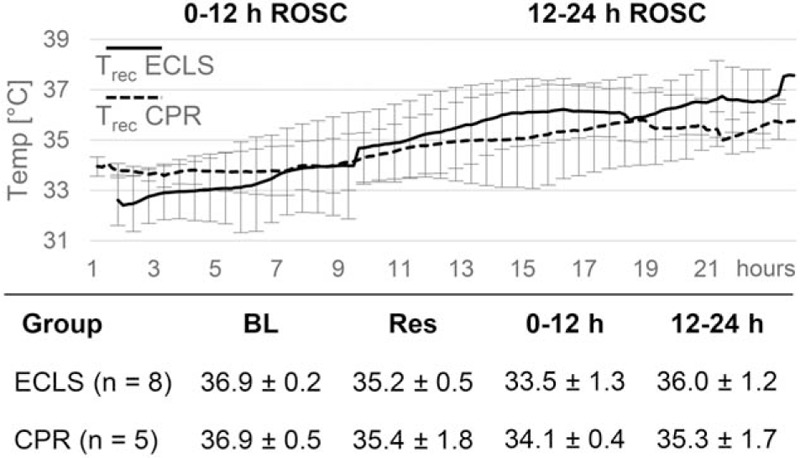
Mild hypothermia as measured intraperitoneally for 12 h after return of spontaneous circulation (ROSC) before 12 h of slow rewarming is depicted.

For hemodynamic and blood sample measurements see Figure 2 and Table 1. Baseline variables were alike in both the groups. ECLS produced higher perfusion pressures and lower etCO_2_ values than CPR. Following ROSC, ECLS animals retained higher MAP and pO_2_. Metabolic changes, including significant decreases of pH, BE, HCO_3_, and increases of lactate and glucose, uniformly occurred in both groups, but electrolyte levels and hematocrit differed between groups. Temperature profiles (Fig. 3) were identical in both the groups.

Temperature measurements (°C) are presented as mean and standard deviation during base line (BL; n = 8 in the CPR group), resuscitation phase (Res; n = 8 in the CPR group) and hours 0 to12 and 12 to 24 after ROSC.

### Outcome

**Fig. 4:**
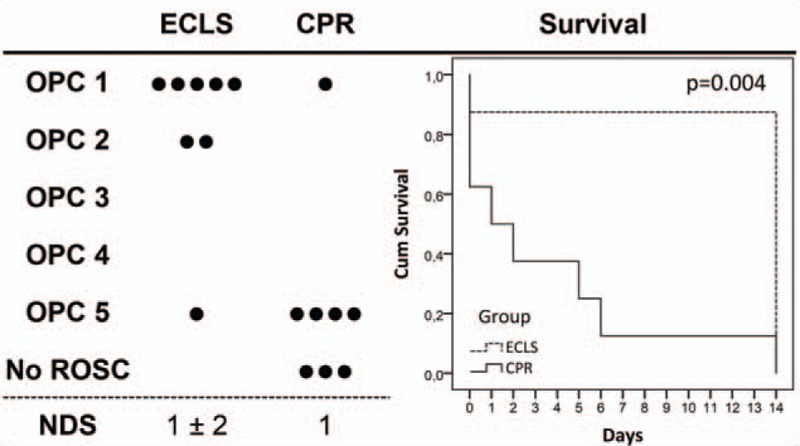
Outcome at 14 days in terms of final overall performance categories (OPC: 1 = normal; 5=dead; ROSC: restoration of spontaneous circulation; each dot represents one rat), neurologic deficit scores (NDS; 0 = normal, 100 = dead, mean and standard deviation), and plotted as Kaplan–Meier curve with log-rank test for group comparison.

**Fig. 5:**
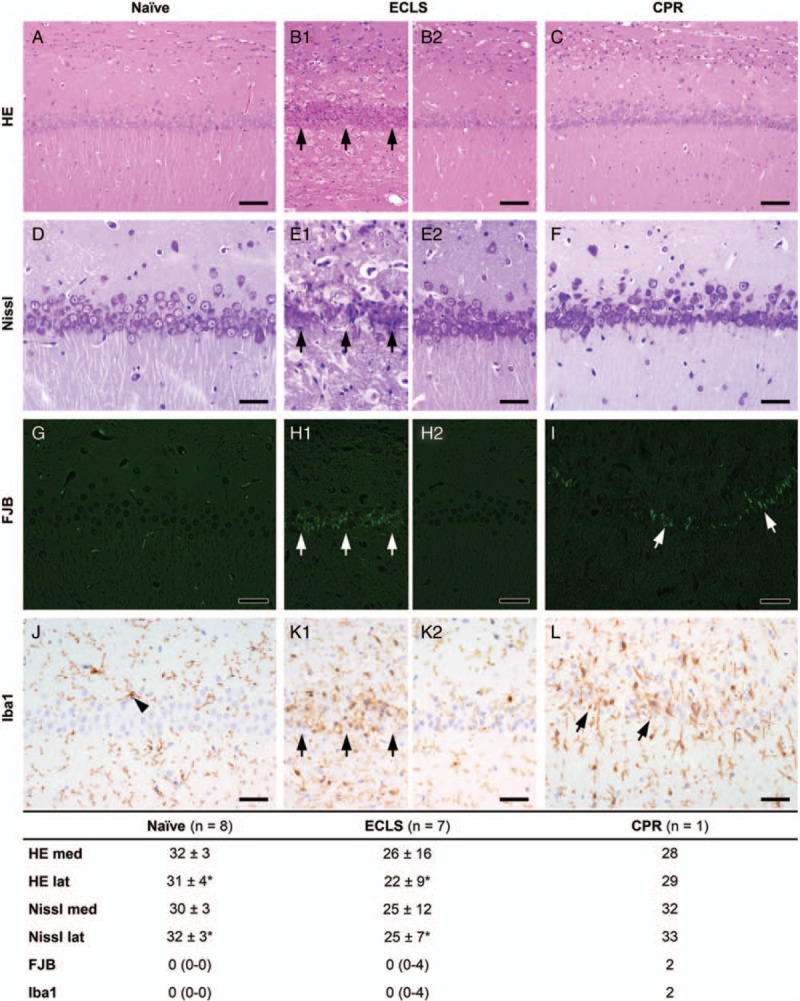
Neuropathologic damage in the hippocampal CA1 region.

Survival data are presented in Figure 4. ROSC was achieved in all the rats. However, ROSC sustained in eight of eight (100%) ECLS and five of eight (63%) CPR animals (*P* = ns). Three CPR rats suffered rearrest after 11 ± 3 min of spontaneous circulation. One of 8 (13%) ECLS and 4 of 5 (80%) CPR rats died after transferral to the cage (*P* = 0.032). The ECLS and one CPR rat died overnight, three CPR rats had to be sacrificed for humane endpoint met: two within the first 2 days because of severe dyspnoea and pain unresponsive to supportive therapy, one rat on day six because of necrotic hind legs. Overall survival at 14 days was 7 of 8 (88%) ECLS rats, compared with 1 of 8 (13%) CPR rats (P = 0.010). The median survival time for ECLS was 14 days (IQR 14–14 days), and 1 day (IQR 0–5 days) for the CPR group, the log-rank test revealed a statistically significant difference between survival over time (*P* = 0.004). Neurologic function was good in all surviving ECLS animals (OPC 1 and 2). Histologic outcome was heterogenic (Fig. 5). Overall living neuron count in the lateral hippocampal CA1 region of ECLS rats was lower compared with naive animals. FJB and Iba1 staining showed damaged neurons and microglial activity in three of the seven ECLS (one grade 2, two grade 4). The only surviving CPR animal had a good functional neurologic outcome and a living neuron count similar to naive animals, while some damaged neurons and microglial activity in FJB and Iba1 staining (grade 2) in the very medial portion of the CA1 were present.

Hematoxylin and eosin (HE) (A–C, bar = 80 μm) and Nissl (D–F, bar = 40 μm), count of living neurons in the medial and lateral CA1 region (mean and standard deviation): A and D, naive animal with normal CA1 region; B1 and E1, ECLS animal with degenerating neurons after cardiac arrest (arrows); B2 and E2, ECLS animal with normal CA1 region; C and F, the only surviving CPR animal with normal CA1 region. Fluoro-Jade-B (FJB) (G to I, bar = 50 μm), semiquantitative rating of fluorescent dying neurons (0 = normal, 4 = intense; median and interquartile range): G, naive animal with normal CA1 region, the small positive signals are due to autofluorescence of erythrocytes; H1, ECLS animal with degenerating neurons (arrows); H2, ECLS animal with normal CA1 region; I, CPR animal with few degenerating neurons in the very medial portion of CA1 (arrows). Iba1 immunohistochemistry (J–L, bar = 40 μm), semiquantitative rating of microglial activation (0 = normal, 4 = intense; median and interquartile range): J, naive animal with normal microglia (arrowhead); K1, ECLS animal with increased staining intensity and number of microglia (arrows); K2, ECLS animal with normal microglia; L, CPR animal with intensified staining of microglia in the very medial portion of CA1 (arrows). ^∗^*P* < 0.05 comparing ECLS versus naive. CPR indicates cardiopulmonary resuscitation; ECLS, extracorporeal life support.

## DISCUSSION

ECLS improved survival in a rat VFCA model after a prolonged no-flow period. While CPR and ECLS proved equally successful after a 6-min VFCA (11), ECLS was necessary for survival after 10 min of VFCA. We could not demonstrate differences in neurologic or histologic outcome though, as only one CPR rat survived, which had a normal OPC and NDS, and living neuron count in CA1. Even if ROSC is a key-element in the chain of survival and an increased number of experiments would have resulted in a significant difference for ROSC between groups, survival, which finally counts, was significantly different with 88% ECLS rats and only 13% CPR rats (*P* = 0.010). Even the median survival time for ECLS was statistically significant different (*P* = 0.004). We think that from an ethical standpoint to kill more animals just for the purpose to have more animals with ROSC especially in the control group is not justified and not in compliance with ARRIVE guidelines (12). For a sophisticated statistical evaluation with regards to neurologic and histopathologic function more animals would have been needed, but this would have made no sense, if in the first step survival could not be increased. Thus, we have decided to use naive histologic controls for group comparisons to make our conclusions more robust.

While we could restart the heart in all rats, three CPR animals suffered re-arrest but none of the ECLS. Furthermore, we found a significant reduction in death following successful reperfusion. Therefore, ECLS not only sustainably restarts the heart during the short period of resuscitation, but induces beneficial effects lasting longer after ROSC. It might be speculated that ECLS ameliorates reperfusion injury in our model, though whether this is due to better organ perfusion during resuscitation, improved myocardial or pulmonary function after ROSC, or more sustained hypothermia remains to be investigated.

CPR has been shown to provide cardiac output and end-organ perfusion well below baseline (18, 19). While cerebral structures seem to be protected initially, this mechanism fails with increased CA duration (2) and duration of CPR (3). A significant correlation between MAP and cardiac output or brain perfusion has been demonstrated in the rat CPR model (18). Thus, it might be hypothesized that increased perfusion pressures during resuscitation observed in our model correlated with improved end-organ perfusion, responsible at least in part for the better outcome in the ECLS group.

After ROSC, ECLS rats were hemodynamically more stable and had improved pO_2_ blood gas values, indicating better myocardial and pulmonary function. Temperature readily dropped and could be sustained mild hypothermic for 12 h after ROSC in both the groups, suggesting equal protection from reperfusion injury. Electrolyte levels on the other hand strongly differed from baseline as well as between groups. Whether and how altered ionized calcium or potassium levels for example determine myocardial and cerebral function, reperfusion injury and ultimately outcome, remains to be investigated (20).

To our knowledge, a beneficial effect of ECLS in reversing prolonged global ischemia and enabling neurologically good survival compared with CPR has not been demonstrated in a VFCA rat model. Besides Janata et al. (11), only Rungatscher et al. (21) successfully resuscitated 30 of 35 rats (86%) by ECLS after 10 min of untreated VF with survival to 2 h. How difficult to realize and prone to procedural failures a VFCA ECLS model is, and how important it is to analyze and report these technical challenges in order to reduce preventable errors and thus animal use, was recently shown (22). Most ECLS resuscitation models in rats therefore induce CA by asphyxia, deep hypothermia, or cardioplegia. However, cerebral blood flow reperfusion patterns differ with type of CA (23). We therefore believe that a VFCA model is highly relevant to CA in adults, most of which results from VF rather than asphyxia (24). Rat studies of CPR after VFCA on the other hand are numerous. However, survival data of prolonged (≥10 min) insult times are scarce. Some studies did achieve sustained ROSC rates between 70% and 100% (19, 25–27), but experiments were terminated few hours subsequent to ROSC, with survival to 4 h of about 40% in one study (26) and median survival of 5 h in another (27). Only one team described ROSC and survival to 7 days after 12 min of VFCA, reporting hind-limb bilateral spastic paralysis as severe neurologic deficit (28). CPR might restart the heart after prolonged VFCA in rats, but ECLS is necessary for long-time survival and good neurologic recovery.

### Limitations

Our study used a rodent model of VFCA with adult but healthy male rats as study subjects, results may thus not be readily applicable to human CA victims, who are typically older men and women with a medical history of cardiovascular disease. Large animal models mimic the clinical setting best and have long been established in CA research to investigate therapeutic concepts in a bench-to-bedside approach (9, 10). A rodent model retains basic human anatomic and physiologic properties (29). It thus adds to the field of research, replacing and reducing the need for large experimental animal research, and refining outcome measures by introducing molecular and immunohistochemical tools to investigate mechanisms of ischemic and reperfusion damage (25).

Animals in this study were not randomized to the two treatment arms. Baseline physiologic parameters, including weight, hemodynamic, temperature, and blood sample values, were similar in both groups, rendering the two groups comparable. Sevoflurane exposure before VFCA, having neuroprotective properties (30), was matched between groups despite longer preparation for the ECLS setup. Although not blinded, uniformity of clinical and neurologic evaluation of all rats—including decision of early termination for humane endpoint of three CPR rats—was ensured by standardized protocols (15–17) and international laboratory animal ethical guidelines (13, 14). To our surprise, the reasons for termination of experiments can usually be related to ECLS treatment, but were only observed in the control group and thus prove in our opinion that ECLS might be beneficial not only for ROSC but also for survival throughout the post-resuscitation period. The decision about endpoints was made according to an *a priori* defined strict protocol. It was usually initiated by laboratory technicians, animal healthcare providers, and veterinarians of the Division of Biomedical Research. Finally, a consensus of the investigator group was established. As such, termination of an experiment was not an easy choice for all involved, considering the already invested spirit, manpower, and financial resources, nonetheless complying with good scientific practice and animal welfare demands. Therefore, we think that we have proven that ECLS improves survival compared with the control group. Blinding for termination and outcome decisions was not possible due to the different surgical procedures in the studied animal groups. Histopathologic outcome was evaluated blinded by a veterinary histopathologist. Degeneration and subsequent recovery of neurons following ischemia is a time dynamic process and therefore requires histologic evaluation at defined same time points after the insult to make investigated groups comparable (31). Routine plasma analysis unfortunately did not include specific ischemic brain damage-related parameters.

Both the CPR and ECLS groups of prolonged VFCA are attractive models to investigate mechanisms and therapies of global cerebral ischemia and reperfusion injury. However, rats with unfavorable outcome either died or had to be sacrificed due to systemic disease. It seems that failure of the organism preceded neurologic impairment, causing a loss of neurologically damaged rats that would also have suffered considerable neuronal loss. In the ECLS cohort on the other hand, significant histological damage was found only in the medial portion of the hippocampal CA1 region, and neurologic outcome consistently favorable. In order to reduce the number of experimental animals, refined methods to monitor cerebral functional outcome have thus been developed based on this model, such as brain metabolic measurements without the need for long-term survival (32, 33). Tests of cognitive function, like Morris water maze, further differentiating functional outcome in surviving animals, are currently being investigated (34).

## CONCLUSION

In conclusion, ECLS after 10 min of VFCA results in 100% resuscitability and favourable functional long-term outcome compared to conventional CPR. This might be explained by increased perfusion pressures during resuscitation, and involve effects lasting longer after ROSC, such as improved cardiopulmonary function.

## Figures and Tables

**Table 1:** Arterial blood samples taken before the experiment (BL) and 5 and 15 min after return of spontaneous circulation presented as mean and standard deviation

	ECLS BL (n = 8)	CPR BL (n = 8)	ECLS 5 min (n = 8)	CPR 5 min (n = 8)	ECLS 15 min (n = 8)	CPR 15 min (n = 5)
pO2 (mm Hg)	142 ± 30	139 ± 29	350 ± 108^*^^,^^†^	158 ± 67^*^	443 ± 68^*^^,^^†^	284 ± 149^*^
pCO2 (mm Hg)	40 ± 6	41 ± 4	54 ± 24	51 ± 18	38 ± 8	46 ± 13
pH	7.40 ± 0.04	7.39 ± 0.03	7.04 ± 0.13^†^	7.02 ± 0.13^†^	7.16 ± 0.08^†^	7.10 ± 0.05^†^
BE (mEq/L)	0 ± 3	0 ± 2	−16 ± 3^†^	−17 ± 3^†^	−14 ± 4^†^	−14 ± 4^†^
HCO3 (mEq/L)	25 ± 3	24 ± 2	12 ± 2^†^	11 ± 2^†^	13 ± 3^†^	17 ± 9
Lac (mmol/L)	1.0 ± 0.3^*^	1.5 ± 0.5^*^	12.1 ± 2.7^†^	12.4 ± 1.6^†^	9.8 ± 3.7^†^	9.3 ± 1.0^†^
Na (mmol/L)	138 ± 3	138 ± 3	142 ± 2^*^^,^^†^	149 ± 4^*^^,^^†^	141 ± 2	143 ± 4
K (mmol/L)	4.1 ± 0.7	4.3 ± 0.3	4.7 ± 0.3^*^^,^^†^	4.1 ± 0.5^*^	3.8 ± 0.7	3.5 ± 0.2^†^
Ca (mmol/L)	1.17 ± 0.12	1.24 ± 0.06	1.34 ± 0.04^*^^,^^†^	1.03 ± 0.12^*^^,^^†^	1.24 ± 0.05^*^	1.03 ± 0.23^*^
Hct (%)	41 ± 5	45 ± 3	32 ± 4^*^^,^^†^	37 ± 6^*^^,^^†^	38 ± 3	44 ± 8
Glu (mg/dL)	150 ± 0	175 ± 33	318 ± 29^*^^,^^†^	257 ± 41^*^^,^^†^	324 ± 30^†^	282 ± 47^†^

^*^P < 0.05 comparing ECLS to CPR.

^†^*P* < 0.05 comparing 5 min or 15 min to BL. CPR indicates cardiopulmonary resuscitation; ECLS, extracorporeal life support.
